# Shifts with Nights and Migraine Prevalence Among Nurses: A Systematic Review and Meta-Analysis

**DOI:** 10.3390/healthcare14060774

**Published:** 2026-03-19

**Authors:** Piedad Gómez-Torres, Azahara Ruger-Navarrete, Laura Lasso-Olayo, Isabel Blázquez-Ornat, David Peña-Otero, Sergio Galarreta-Aperte

**Affiliations:** 1 Department of Nursing, Faculty of Health Sciences of Ceuta, University of Granada, 51001 Ceuta, Spain; azahara.ruger@ugr.es (A.R.-N.); sgalarreta@ugr.es (S.G.-A.); 2 SAPIENF Research Group (B53_23R), University of Zaragoza, 50009 Zaragoza, Spain; lasso@unizar.es (L.L.-O.); iblazquez@unizar.es (I.B.-O.); 3 Department of Physiatry and Nursing, Faculty of Health Sciences, University of Zaragoza, 50009 Zaragoza, Spain; 4 Marqués de Valdecilla University Hospital, Cantabrian Health Service, 39000 Santander, Spain; david.penao@scsalud.es; 5 Research Group of Nursing in Instituto de Investigación Sanitaria Valdecilla Marqués de Valdecilla (IDIVAL), 39011 Santander, Spain

**Keywords:** migraines, nurses, Shift Work Schedule, occupational health, occupational diseases

## Abstract

**Highlights:**

**What are the main findings?**

- In a nurse-specific systematic review and meta-analysis (4 observational studies; total N = 3843, analyzable N = 3323), night-inclusive schedules (fixed nights and/or rotating shifts including nights) were not associated with a statistically significant difference in 1-year migraine prevalence versus day-only/non-night schedules (pooled PR = 0.95, 95% CI 0.82–1.10; I^2^ = 0%).
- Secondary night-work intensity comparisons were inconclusive (high vs. low: PR = 1.24, 95% CI 0.46–3.36; high vs. zero nights: PR = 0.85, 95% CI 0.38–1.93), with non-harmonized exposure thresholds and few contributing studies limiting precision and consistency.

**What are the implications of the main findings?**

- Current nurse-specific evidence does not support strong conclusions or roster-policy recommendations based on migraine prevalence alone, because the evidence base is small, mostly cross-sectional, and relies on heterogeneous exposure definitions and crude PRs.
- Future research should use prospective designs that distinguish fixed night work from rotating-with-nights schedules and measure shift-pattern features (e.g., irregularity, quick returns, consecutive nights), while assessing sleep/circadian mediators and migraine outcomes beyond prevalence (incidence, attack frequency, chronification, disability, medication use).

**Abstract:**

**Background**: Fixed night work and rotating schedules including nights may contribute to migraine via sleep disruption and circadian misalignment, but evidence is inconsistent and definitions vary. This systematic review and meta-analysis compared past-year migraine prevalence in nurses working night-inclusive schedules versus day-only or non-night schedules. **Methods**: Following PRISMA 2020 and registered in PROSPERO (CRD420261304288), we searched PubMed, Scopus, Web of Science, CINAHL, and the Cochrane Library from inception to 3 February 2026 (English/Spanish). Observational studies in nurses (≥18 years) reporting past-year migraine prevalence by shift pattern were eligible. All included studies assessed past-year prevalence; pooled PRs reflect 1-year prevalence. Crude prevalence ratios (PRs) were calculated from contingency tables and pooled quantitatively. Risk of bias was assessed with the JBI prevalence checklist. **Results**: We identified 54 records; 4 studies were included (N = 3843) of which 3323 participants contributed to the comparative meta-analysis because complete disaggregated data were available to construct contingency tables. The pooled association between night-inclusive schedules and migraine prevalence was not statistically significant (PR = 0.95, 95% CI 0.82–1.10; I^2^ = 0%). Secondary intensity contrasts were inconclusive (high vs. low: PR = 1.24, 95% CI 0.46–3.36; high vs. zero nights: PR = 0.85, 95% CI 0.38–1.93). **Conclusions**: Current nurse-specific evidence does not show a statistically significant difference in migraine prevalence between night-inclusive and non-night schedules; however, the small evidence base and limited generalizability preclude firm conclusions. Future longitudinal studies are needed to clarify this association.

## 1. Introduction

Migraine is a primary neurological disorder characterized by recurrent headache attacks, often accompanied by nausea, photophobia, and phonophobia, and may be associated with substantial functional impairment. Its clinical definition and diagnostic classification are based on the International Classification of Headache Disorders (ICHD-3), which is widely used in research and clinical practice [1]. From a public health perspective, migraine is one of the leading causes of nonfatal health loss worldwide: analyses from the Global Burden of Disease (GBD) rank it as the second leading global cause of years lived with disability (YLDs) and the leading cause among young women, underscoring its impact on the working-age population [2,3]. Recent epidemiological reviews have further reinforced the high global prevalence of migraine and its substantial public health burden [4].

Various factors can trigger or modulate migraine attacks, and there is growing interest in the role of sleep and circadian rhythms. Studies and reviews have reported that attack onset may follow circadian variation patterns, consistent with the hypothesis of an underlying chronobiological susceptibility [5,6]. Likewise, sleep deprivation, sleep fragmentation and misalignment of the sleep–wake cycle have been associated with a higher migraine burden and with plausible pathophysiological mechanisms, including alterations in pain modulation and the trigeminovascular system [7,8,9]. Recent real-world data also suggest that sleep fragmentation and deviations from habitual sleep patterns may be associated with next-day migraine occurrence, further supporting a bidirectional relationship between sleep and migraine [9].

In this context, work environments that promote sleep displacement and circadian misalignment are particularly relevant. Shift work, particularly fixed night work or rotating schedules that include nights, is associated with sleep disruption and circadian misalignment and is especially common in nursing because of the need for continuous care [10,11,12,13]. Recent reviews and meta-analyses have linked shift work, irregular night shifts, and night work to headache and migraine risk, although the evidence remains heterogeneous and not all studies isolate the specific contribution of rotating or irregular schedules [14,15,16]. Nurse-specific studies also suggest a substantial burden of headache disorders in this workforce [17,18]. Prospective evidence indicates that insomnia, shift work disorder, and restless legs syndrome may predict future headache and migraine more consistently than work schedule characteristics alone [19], whereas acute within-person data have shown a higher prevalence of headache on night-shift days than on day-shift days [20]. However, the available literature remains difficult to interpret because exposure definitions are heterogeneous, and studies often group fixed night work together with rotating schedules that include nights, limiting the ability to isolate the specific effects of irregular or rotating patterns [14,15,16].

This uncertainty is clinically relevant because, if night work or night-inclusive shift schedules are associated with a higher prevalence or risk of migraine, preventive and organizational strategies could be warranted in an essential healthcare workforce. Clarifying this association is particularly relevant in nursing because night-inclusive schedules are common and migraine may affect well-being, work functioning, and workforce sustainability. However, despite the high prevalence of night-inclusive schedules in nursing, the evidence remains fragmented and no nurse-specific quantitative synthesis has clearly summarized past-year migraine prevalence differences by shift pattern. This represents an important research gap, because previous reviews have generally combined heterogeneous occupational groups or broader shift-work categories. Although the present study does not test a novel intervention, its innovative contribution lies in applying a nurse-specific and circadian-health perspective to migraine prevalence, thereby linking neurological outcomes with occupational well-being and potentially modifiable organizational factors in healthcare work. Therefore, this systematic review and meta-analysis aimed to compare past-year (1 year) migraine prevalence in nurses working night-inclusive schedules (fixed night work and/or rotating schedules including nights) versus day-only or non-night schedules.

## 2. Materials and Methods

This study is a systematic review with meta-analysis conducted in accordance with the Preferred Reporting Items for Systematic Reviews and Meta-Analyses (PRISMA 2020) [21] and was registered in PROSPERO, under registration number CRD420261304288 [22].

### 2.1. Study Design

A systematic review of observational studies with meta-analysis was conducted. The question was structured using PICO: P: adult nurses in healthcare settings; I/E: exposure to night shift and/or rotating shifts that include nights; C: fixed day shift without nights (when available); O: prevalence of migraine.

### 2.2. Information Sources and Search Strategy

Systematic searches were conducted in CINAHL Complete, the Cochrane Library, PubMed, Scopus, and Web of Science. The search strategy was developed collaboratively by two investigators and adapted to the specifications of each database; however, no formal external validation or librarian/information specialist review was performed. The search was limited to publications in English or Spanish, with no date restrictions, and was completed on 3 February 2026. In addition, manual reference checking (backward citation searching) was performed for the included studies and relevant reviews.

The following search strategy was used, adapted to the specifications of each database:

((“Nurses”[MeSH Terms] OR nurse*[tiab] OR “nursing staff”[tiab] OR “registered nurse”[tiab] OR “registered nurses”[tiab]) AND (“Shift Work Schedule”[MeSH Terms] OR shift work*[tiab] OR night shift*[tiab] OR “night shift”[tiab] OR “night shifts”[tiab] OR rotating shift*[tiab] OR “rotating shift”[tiab] OR “rotating shifts”[tiab] OR night work[tiab] OR nocturn*[tiab] OR rotat*[tiab]) AND (“Migraine Disorders”[MeSH Terms] OR migraine*[tiab] OR “primary headache disorder”[tiab] OR “primary headache disorders”[tiab] OR “headache disorder”[tiab] OR “headache disorders”[tiab])).

### 2.3. Eligibility Criteria

#### 2.3.1. Inclusion Criteria

Observational studies (cross-sectional and cohort) conducted in adult nurses (≥18 years) working in healthcare settings with scheduled shifts were included. Eligible professional profiles comprised registered nurses, licensed practical/vocational nurses, and nursing staff. Studies with mixed samples of healthcare professionals were included only if nursing-specific data could be extracted separately.

The exposure of interest was night shift work and/or rotating shifts that included nights. Night shift was defined as any schedule with ≥3 h between 22:00 and 06:00, or the closest operational definition reported by the study. Exposure intensity (e.g., number of nights per month/period or duration in shift work) was extracted when available.

The primary outcome was migraine prevalence, using the following ascertainment methods: (1) ICHD criteria or a clinical diagnosis; (2) validated screening instruments; or (3) a clearly defined self-report.

When available, the comparator was a fixed day schedule without night work; however, studies were not excluded due to the absence of a comparator (prevalence-only studies were eligible). The prevalence timeframe (point, 12-month, lifetime/‘ever’) was extracted. In this review, all included studies reported past-year (1-year) migraine prevalence; therefore, pooled PRs refer to 1-year prevalence and no cross-timeframe harmonization was required.

#### 2.3.2. Exclusion Criteria

The following were excluded: participants < 18 years; samples composed exclusively of students who were not working as nurses; studies with mixed occupational samples in which nursing data were not separable; and studies without extractable migraine data. Trials or other experimental/interventional designs, case reports/series without a population denominator, editorials, commentaries, letters, narrative reviews, protocols, and conference abstracts/reports with insufficient information were also excluded.

In addition, studies were excluded when the exposure was daytime work only (without nights), on-call duties without a defined pattern of night/rotating shift work, or when exposure was insufficiently described and could not be classified (night vs. rotating with nights vs. day) or was not extractable at the nursing-specific level.

### 2.4. Study Selection and Data Extraction

Records retrieved were managed in the Zotero^®^ Version 8.0.1 reference manager to remove duplicates. Study selection was performed in two phases: (1) title/abstract screening and (2) full-text assessment, applying the predefined eligibility criteria. Two reviewers completed the process independently; discrepancies were resolved by consensus and, if they persisted, by a third reviewer.

Two reviewers independently extracted and cross-checked the data. We did not contact study authors for additional information. This decision was made a priori due to feasibility constraints and to avoid selective availability of unpublished data; its potential impact on eligibility and completeness of contingency tables is acknowledged as a limitation. Data were extracted using a pre-specified form, including:

1. Study characteristics: author/year, country, design, setting/clinical unit, sample size, response rate/sampling method.
2. Population characteristics: age (mean/range), proportion of women, study inclusion criteria, relevant variables (e.g., tenure).
3. Exposure: shift type (fixed night vs. rotating with nights), operational definition, frequency/intensity (nights/month or categories), exposure duration if reported.
4. Outcome: migraine ascertainment method (ICHD/clinical diagnosis/validated instrument/self-report), time frame (point/12-month/lifetime), numerator and denominator (x/n).
5. Comparison (if available): group-specific data (night/rotating vs. day), adjusted effect estimates (OR/RR/PR) and covariates included in the adjustment.

**Figure 1:**
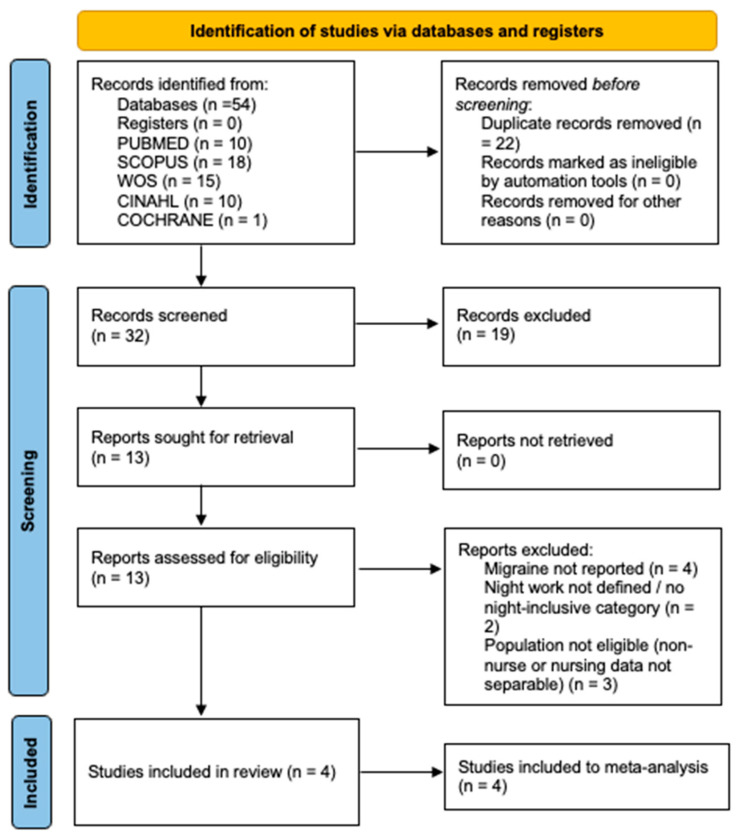
PRISMA flow diagram of the study selection and inclusion process.

When a study reported multiple estimates (e.g., several categories of night-shift frequency), the comparison of any shift including nights versus day shift was prioritized; for dose–response analyses, the original categorization was extracted and, when feasible, harmonized into predefined subgroups (e.g., high vs. low exposure, or “>X nights” vs. “≤X nights”). Reasons for full-text exclusion were recorded using prespecified categories and are reported in the PRISMA flow diagram (Figure 1). In this review, all included studies provided comparable group-level data to derive crude PRs for night-inclusive schedules versus day/no-night schedules.

### 2.5. Risk of Bias

Methodological quality (risk of bias) of prevalence studies was assessed using the JBI Critical Appraisal Checklist for Studies Reporting Prevalence Data [23] (9 items). Each item was rated as Yes/No/Unclear/NA and, in line with JBI recommendations, no total score was calculated; instead, an overall judgment was issued for each study (Include/Exclude/Seek further info) based on the pattern of responses. Items related to representativeness and selection (items 1–2) and to the validity/standardization of condition measurement (items 6–7) were considered critical; a No on any of these led to Exclusion or, if information was insufficient, Seek further info. To describe residual limitations among included studies, an operational criterion was applied: minor concerns (only Unclear in non-critical items), moderate concerns (≥2 No/Unclear in non-critical items or item 8 rated No/Unclear), and major concerns (No in critical items or unaddressed non-response issues).

### 2.6. Data Analysis

A meta-analysis was conducted in R (RStudio 2025.09.2 + 418 for macOS) using the meta and metafor packages, based on study-level contingency tables (migraine yes/no among those exposed to night-inclusive schedules vs. day shifts or shifts without nights). The effect measure was the crude prevalence ratio (PR); analyses were performed on log(PR) using inverse-variance weighting and standard errors derived via an asymptotic approximation. There were no zero-count cells; therefore, no continuity corrections were applied.

Given expected conceptual heterogeneity across studies and the limited informativeness of heterogeneity estimates when k = 4, the primary analysis was conducted using a random-effects model (τ^2^ estimated by REML) with Hartung–Knapp confidence intervals (ad hoc correction). A common-effect model was examined as a sensitivity analysis. Heterogeneity was assessed using Q, I^2^, and τ^2^, and results were presented in forest plots with total and analyzable sample sizes. Robustness was evaluated through leave-one-out analyses and influence/outlier diagnostics implemented in the metafor package.

As secondary analyses, night-shift frequency was compared using a within-study “high vs. low” approach (highest vs. lowest available category) and a “high vs. 0 nights” contrast when a 0-night reference was available, estimated primarily with random-effects models (REML + Hartung–Knapp).

## 3. Results

### 3.1. Search Results

The literature search identified 54 records in total: PubMed (n = 10), Scopus (n = 18), Web of Science (n = 15), CINAHL (n = 10), and Cochrane (n = 1). After removing 22 duplicates, 32 unique records were screened by title and abstract, of which 13 underwent full-text assessment. After full-text review, 4 studies met the eligibility criteria and underwent methodological quality assessment to determine inclusion in the synthesis. Ultimately, 4 studies were included in the systematic review and meta-analysis. The study selection process is illustrated in Figure 1.

### 3.2. Characteristics of the Included Studies

Four studies (n = 3843) were included, providing sufficient data to estimate crude prevalence ratios (PRs) for migraine comparing night shifts (or rotating shifts including nights) versus day shifts [17,18,24,25].

Three were cross-sectional studies: one conducted in Norway within a cohort of nurses (wave 6 survey in 2014; n = 1585), with migraine identified using an ICHD-IIIb–based questionnaire and participants classified by work schedule (shifts including nights vs. day shifts or shifts without nights) [17]; and two conducted in China, one in Northern China (December 2013–June 2014) in three tertiary 3A hospitals, using cluster sampling and neurologist confirmation (by telephone when applicable), with n = 1023 nurses and ICHD-3 beta criteria [24]; and another in Southern China (Sanya, May–October 2018) in three tertiary A hospitals and one secondary A hospital, in which 548 healthcare professionals responded (including 308 nurses), and the diagnosis was confirmed through neurologist interviews according to ICHD-3 [25]. The fourth study was retrospective, based on the occupational health surveillance database of a large hospital in Italy (Vito Fazzi Hospital, Lecce; March 2024–February 2025), and included 975 registered nurses, with migraine diagnosed according to ICHD-3 beta [18].

Although the aggregated sample size was 3843, the analyzable N for the comparative meta-analysis based on contingency tables was 3323, corresponding to participants with complete, disaggregated information on (i) exposure (shifts including nights vs. day shifts or shifts without nights) and (ii) outcome (migraine yes/no). This information was required to construct contingency tables and calculate crude PRs.

Regarding methodological quality, all studies met the core items of the appraisal tool, although some raised concerns in specific domains, mainly due to incomplete reporting for certain variables and minor methodological limitations. Accordingly, no study was excluded based on quality, and the assessment was used to contextualize interpretation of the findings. Item-level risk-of-bias judgments for each study using the JBI prevalence checklist are provided in Appendix A.

Across studies, the main concerns related to sample representativeness (hospital-based samples), incomplete reporting for some exposure strata, and residual confounding given that pooled estimates were based on crude contingency tables. Migraine ascertainment was ICHD-based and referred to the past year in all included studies, supporting outcome validity. Response rates in the cross-sectional surveys ranged from 69.4% to 93.0%, while the retrospective occupational surveillance study does not provide a conventional survey response rate. Most samples were hospital-based and exposure definitions varied (fixed night vs. rotating-with-nights and intensity thresholds), which may limit representativeness and contribute to exposure misclassification [17,18,24,25].

### 3.3. Meta-Analysis Results

#### 3.3.1. Primary Analysis

**Figure 2:**
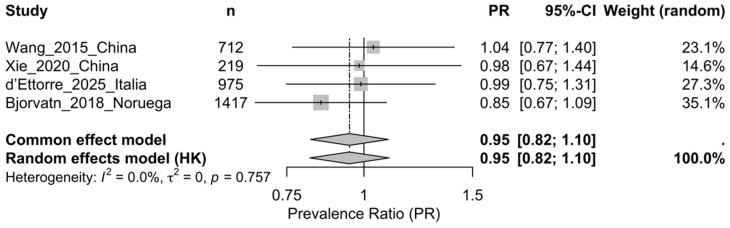
Forest plot of the migraine prevalence ratio (PR) for shifts including nights versus fixed day shifts without nights [17,18,24,25]. Note: The primary estimate was obtained using a random-effects model (REML + Hartung–Knapp, ad hoc correction); a common-effect model is shown as a sensitivity analysis. Weights correspond to the random-effects model.

In the primary analysis (random-effects model; τ^2^ by REML; Hartung–Knapp CI with an ad hoc correction), the association was not statistically significant: PR = 0.95 (95% CI 0.82–1.10; *p* = 0.33). The between-study variance estimate was τ^2^ = 0; however, with k = 4, low I^2^/τ^2^ values should be interpreted cautiously because the meta-analysis had limited power to detect between-study variability (Figure 2).

Because no zero-count cells were observed, no continuity correction was applied. Statistical heterogeneity was not detected (Q = 0.85, df = 3; *p* = 0.757; I^2^ = 0%; τ^2^ = 0), although with k = 4 these estimates should be interpreted cautiously because power to detect between-study variability is limited. Leave-one-out analyses showed stability of the pooled effect (PR 0.92–1.00). Influence diagnostics identified Bjorvatn as the most influential study [17]; in a targeted sensitivity analysis excluding it, the effect remained non-significant (PR = 1.00, 95% CI 0.84–1.20; *p* = 0.97; I^2^ = 0%; τ^2^ = 0).

Because pooled estimates are based on crude contingency tables, they should be interpreted as unadjusted associations; potential confounding factors such as age, workload, sleep quality, and chronotype were not accounted for in the pooled analysis and may bias the PR in either direction.

#### 3.3.2. Category-Based Analyses

To explore whether the association varied by night-work intensity, we conducted predefined category-based analyses using the night-frequency strata reported within each study. These analyses were considered secondary/exploratory because thresholds defining “high” and “low” exposure (and the availability of a “zero nights” reference) were not harmonized across studies, and the number of contributing studies per contrast was small. Accordingly, pooled estimates were derived using random-effects models (REML with Hartung–Knapp confidence intervals, ad hoc correction) and were interpreted primarily in terms of direction, consistency, and heterogeneity rather than statistical significance alone. Definitions of ‘high’, ‘low’, and ‘zero-night’ categories are summarized in Table 1.

Only studies providing sufficient stratum-level information to compute crude PRs contributed to PR-based pooling for the intensity contrasts. Wang et al. did not contribute because night-shift frequency strata were reported as prevalences (%) and ORs without stratum denominators.

- High frequency vs. low frequency (k = 3)

**Figure 3:**
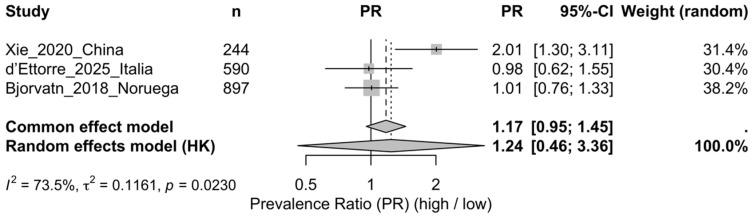
Forest plot of the migraine prevalence ratio (PR) comparing high versus low night-work frequency (highest vs. lowest available category within each study) [17,18,25]. Note: Pooled estimate obtained using a random-effects model (REML) with Hartung–Knapp confidence interval (ad hoc correction). Heterogeneity is reported as Q, I^2^, and τ^2^; weights correspond to the random-effects model.

In the random-effects model (REML + Hartung–Knapp), the association was inconclusive: PR = 1.24 (95% CI 0.46–3.36), with substantial heterogeneity (Q = 7.55, df = 2, *p* = 0.023; I^2^ = 73.5%; τ^2^ = 0.116). Xie showed a higher prevalence in the high- versus low-frequency group (PR = 2.01; 95% CI 1.30–3.11) [25], whereas d’Ettorre [18] and Bjorvatn [17] were compatible with no differences (95% CI includes 1), suggesting between-study inconsistency and limiting the robustness of the pooled effect (Figure 3). Small-study effects (funnel plot/Egger test) were not assessed due to the insufficient number of studies.

B. High frequency vs. 0 nights (k = 2)

**Figure 4:**
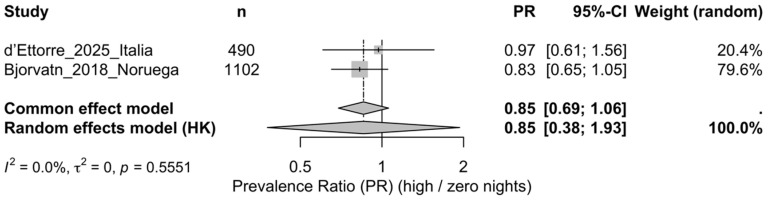
Forest plot of the migraine prevalence ratio (PR) comparing high night-work frequency versus zero nights (when a 0-night reference category was available) [17,18]. Note: Pooled estimate obtained using a random-effects model (REML) with Hartung–Knapp confidence interval (ad hoc correction). With k = 2 studies, heterogeneity statistics (Q, I^2^, τ^2^) are of limited informativeness. The operational definition of “high” differed between studies (>5/month vs. >20/year), so results should be interpreted cautiously; weights correspond to the random-effects model.

In the random-effects model (REML + Hartung–Knapp), no conclusive differences were observed: PR = 0.85 (95% CI 0.38–1.93). No statistical heterogeneity was detected (Q = 0.35, df = 1, *p* = 0.555; I^2^ = 0%; τ^2^ = 0), although with k = 2 heterogeneity assessments are not very informative. The definition of “high” differed across studies (>5/month and >20/year), so results should be interpreted with caution (Figure 4).

## 4. Discussion

In this systematic review and meta-analysis (k = 4 observational studies), we did not detect a difference in 1-year migraine prevalence between nurses working night-inclusive schedules and those working day-only/non-night schedules (pooled PR = 0.95, 95% CI 0.82–1.10). This finding should be interpreted as evidence of limited precision, not as evidence of no association. With only four studies, the meta-analysis has limited ability to characterize true between-study variability, and the confidence interval remains compatible with both a modest decrease and a modest increase in prevalence. Although heterogeneity statistics were low (I^2^ = 0%; τ^2^ = 0), these estimates should be interpreted cautiously when k is small because power to detect between-study variability is limited, and they do not rule out clinically relevant differences across specific shift patterns or settings.

Secondary analyses suggest that, rather than the simple “nights vs. days” dichotomy, the intensity and/or configuration of exposure may be more relevant. In the within-study contrast of “high vs. low” night-shift frequency (k = 3), the pooled effect was imprecise and inconclusive (PR = 1.24; 95% CI 0.46–3.36), with substantial heterogeneity (I^2^ = 73.5%) and discordant directions across studies. In the “high vs. 0 nights” contrast (k = 2), no conclusive differences were observed either (PR = 0.85; 95% CI 0.38–1.93). Overall, these findings suggest that, if an effect exists, it may be concentrated in specific components of shift patterns (e.g., irregularity, rapid rotation, or insufficient rest periods), and that non-uniform definitions of “high exposure” may contribute to the observed variability.

Importantly, our outcome was prevalence (past-year migraine status), which integrates incidence and disease duration and may be less sensitive than alternative endpoints to shift-related sleep and circadian disruption. Therefore, the absence of a detectable prevalence difference does not exclude effects on outcomes such as incident migraine, attack frequency, chronification, disability, medication use, or healthcare utilization, which may respond more directly to sleep disruption and workload dynamics.

From an occupational health perspective, these results provide a nurse-specific quantitative summary of the currently available prevalence data; however, they do not justify strong scheduling recommendations on their own. Any organizational implications should be framed cautiously given the small evidence base, the predominance of cross-sectional designs, and heterogeneity in how night-inclusive exposure was defined (fixed nights vs. rotating-with-nights; intensity thresholds). Future studies with more granular exposure measurement are needed before drawing practice-oriented conclusions about specific roster policies.

Generalizability is limited. Included studies were hospital-based and conducted in three countries (China, Norway, and Italy) with potentially different staffing models, roster practices, and regulatory contexts; samples were predominantly female and may not represent other nursing populations (e.g., community care, long-term care, mixed-sex cohorts, or systems with different night-rotation norms and recovery periods). Additionally, combining fixed night work and rotating schedules including nights under a single exposure construct may dilute pattern-specific effects and constrain applicability to settings where these schedules are clearly separable.

In the included studies, multivariable analyses (reported as adjusted ORs) were broadly consistent with the crude PR findings in that work schedule/night-work exposure was not retained as an independent correlate of migraine. In Bjorvatn et al., adjusted logistic regression (sex, age, percentage of full-time equivalent, marital status, and children living at home) showed no association between migraine and work schedule or the number of night shifts in the previous year [17]. In d’Ettorre et al., logistic regression adjusted for age, marital status, and children living at home found no significant association between migraine and work schedule or night-shift frequency [18]. In Xie et al., multivariate logistic regression in nurses did not identify work arrangement as a risk factor for migraine, whereas marital status and job title were retained [25]. In Wang et al., work arrangement was not retained for migraine in multivariable models, while seniority ≥ 5 years was associated with migraine; night-shift frequency was additionally compared descriptively using a median split [24]. However, these adjusted estimates are ORs with heterogeneous covariate sets and are not directly comparable to the crude PRs pooled here.

Our results are consistent with prior reviews describing conflicting evidence on night-inclusive shift work and migraine, with associations that appear to depend on the shift pattern, tenure, specific job tasks, and the number of night shifts per month [14]. This inconsistency may also reflect differences in how exposure is defined (fixed night shifts vs. rotating schedules, intensity, irregularity), how outcomes are measured (ICHD criteria, clinical diagnosis, questionnaires) which complicates direct comparisons across studies and limits generalizability.

External evidence in broader populations suggests that associations between shift work and migraine may depend on the specific shift pattern, particularly rotation or irregularity, and may also vary across populations [15,16,26]. However, these data are not directly comparable to our nurse-specific prevalence-based meta-analysis, which combined fixed night work and rotating schedules including nights under a single exposure category.

From a biological perspective, the association between rotating/irregular shift work and migraine is consistent with the broader framework of circadian disruption and its health consequences. A landmark review describes how circadian disruption is linked to the risk and/or severity of multiple disorders, including neurological and psychiatric conditions [27]. In migraine, temporal patterns in attack onset have also been described, with circadian variation and subgroups showing a tendency toward morning onset, supporting the relevance of biological timing [28]. In this context, frequent rotation may promote persistent circadian misalignment, sleep fragmentation, and greater instability of the pain-regulation system, particularly when shift schedules prevent the consolidation of stable routines and increase the likelihood of chronic sleep restriction.

In addition, rotating/irregular schedules add behavioral and psychosocial exposures that, in the general population, have been linked to migraine. First, stress is widely described as a common trigger for attacks and as an important factor in causal models of migraine [29,30]. Shift rotation may increase demands, interfere with personal life, and promote sustained stress, potentially modulating susceptibility. Second, insufficient sleep and sleep disorders constitute a cross-cutting pathway. In nursing, the burden of sleep problems is high; a recent meta-analysis estimated the prevalence of shift work disorder (SWD) in nurses and assessed associated factors [12]. In a longitudinal study in nurses, SWD has been observed to be associated with a higher risk/frequency of headaches, including migraine, suggesting a possible role as a mediator or effect modifier [19]. Therefore, the relationship between rotating/irregular shift work and migraine may be better understood as a multifactorial model (circadian misalignment + sleep + stress + habits), in which the “irregularity” component amplifies concurrent exposure to multiple factors that can precipitate attacks.

The fact that our meta-analysis did not detect differences in prevalence in nursing does not negate these mechanisms; rather, it suggests that, in this workforce and with the current evidence base, factors may operate that attenuate or mask associations. First, there is the issue of prevalence versus incidence: prevalence is sensitive to disease duration and remission; if exposure to night-inclusive shifts increases attack frequency or disability but not migraine “status” (yes/no), the effect may not be captured by prevalence PRs. This is consistent with clinical literature in which night-inclusive shift work may be associated with chronification or greater disability in individual cases, even if it does not “create” new cases at scale [31]. Second, in nursing, a selection/adaptation component is plausible (e.g., switching to day shifts due to intolerance or more severe migraine), consistent with a “healthy worker effect,” which could reduce observed differences between groups. Third, exposure misclassification is a critical issue: combining “fixed night work” and “rotating schedules including nights” may dilute signals if risk is concentrated in irregularity; likewise, unmeasured features (rapid rotation, quick returns, consecutive nights, or a high number of nights over short periods) may be more relevant than the overall shift label. This is consistent with findings in nurses in which variables such as SWD, insomnia, or a high number of nights per year are more clearly associated with headaches than “shift type” per se [12,19,25].

Although no overall increase in migraine prevalence in nurses was observed for “nights vs. days,” external evidence suggests that prevention efforts may be better focused on how shifts are organized and on identifying intermediate vulnerabilities. First, prioritize rosters that reduce irregularity, rapid rotation, and insufficient rest periods. Second, incorporate screening and management of shift-related sleep disorders (e.g., SWD), given their frequency in nursing and their potential link with headaches [12,19]. These measures are consistent with the literature that identifies circadian disruption and sleep as central nodes in this problem [10,11,27] and could be integrated into occupational health surveillance and health promotion programs.

Strengths and limitations. The main strength of this work is that it provides a synthesis specific to the nursing population, integrating a comparative meta-analysis and robustness analyses (common-effect vs. random-effects models, influence analysis, and leave-one-out). However, several limitations should be considered: (i) the number of included studies was small (k = 4) and the predominant design was cross-sectional, which limits causal inference; (ii) there was variability in the definition and measurement of exposure (type and intensity of shifts) and in migraine diagnostic methods, with potential misclassification; (iii) the meta-analysis was based on crude estimates, so residual confounding cannot be ruled out; and (iv) with so few studies, the assessment of publication bias and small-study effects is necessarily limited and inconclusive.

Future studies should prioritize longitudinal designs in nursing, with detailed characterization of shift patterns (irregularity, rotation, quick returns, consecutive nights), standardized migraine assessment (ICHD), and explicit evaluation of mediators (SWD/insomnia, stress, chronotype, and meal regularity). This would help identify more vulnerable subgroups and clarify whether the true effect manifests in incidence, chronification, frequency, or disability, rather than prevalence.

## 5. Conclusions

In this systematic review and meta-analysis including four observational studies in nursing, we did not detect a statistically significant difference in 1-year migraine prevalence between night-inclusive schedules and day-only/non-night schedules (pooled PR close to unity). However, the available evidence is limited and does not allow firm conclusions about whether night-inclusive work meaningfully affects migraine prevalence in this population. Future prospective studies with more precise exposure definitions and standardized migraine assessment are needed to clarify this association.

## Figures and Tables

**Table 1:** Night-shift frequency strata used for category-based (“intensity”) analyses and data availability for crude PR estimation.

Study	Bjorvatn et al. [17]	d’Ettorre et al. [18]	Xie et al. [25]	Wang et al. [24]
Night shift frequency	Nights/year (0; 1–20; >20)	Nights/month (0; 1–5; >5)	Nights/month (<6; ≥6)	Nights/month (≤8; >8)
Low category migraine cases n (%)	1–20 nights/year: 70/389 (18.0%). Denominator not explicitly reported	1–5 nights/month: 85/485 (17.5%)	<6 nights/month: 22/117 (18.8%)	≤8 nights/month: 18.9% (denominator not reported)
High category migraine cases n (%)	>20 nights/year: 92/508 (18.1%). Denominator not explicitly reported	>5 nights/month: 18/105 (17.1%)	≥6 nights/month: 48/127 (37.8%)	>8 nights/month: 29.4% (denominator not reported)
Zero nights category migraine cases n (%)	130/594 (21.9%)	68/385 (17.7%)	Not available as a separate reference	Not reported as a separate reference
Information reported	Migraine cases (n) and percentage by category	Stratum distribution, cases and percentage	Cases, percentage, OR (2.72 [95% CI 1.43–5.15] for ≥6 vs. <6)	OR (migraine) = 1.79 (95% CI 1.12–2.85) for >8 vs. ≤8, plus prevalences

Notes: Night-shift frequency was categorized as reported in each study (time window: per year or per month). “High” denotes the highest night-shift frequency category and “Low” the lowest available non-zero category (or the lowest available category when a distinct non-zero low category was not separable). “Zero nights” denotes an explicit 0-night reference category when reported. Values are migraine cases/total (%) when stratum totals were provided; otherwise, values are migraine cases (n) and prevalence (%) as reported. In Wang et al., prevalences (%) and odds ratios (ORs) for >8 vs. ≤8 night shifts/month were reported without stratum denominators, which precluded calculation of crude PRs for PR-based pooling in the intensity contrasts [24]. For Bjorvatn et al., stratum denominators were not explicitly reported; when both the number of cases and prevalence (%) were available, denominators were back-calculated as n/(p/100) and rounded to the nearest integer. Because percentages were rounded in the original report, the reconstructed denominators may differ by ±1 [17]. Abbreviations: OR, odds ratio; CI, confidence interval; PR, prevalence ratio.

## Data Availability

No new data were created or analyzed in this study. Data sharing is not applicable to this article.

## Abbreviations

The following abbreviations are used in this manuscript:

CI	Confidence Interval
CINAHL	Cumulative Index to Nursing and Allied Health Literature
df	Degrees of freedom
GBD	Global Burden of Disease
I/E	Intervention/Exposure (within PICO)
ICHD	International Classification of Headache Disorders
ICHD-3	International Classification of Headache Disorders, 3rd edition
ICHD-IIIb/ICHD-3 beta	Beta version of the 3rd edition of the International Classification of Headache Disorders
IHS	International Headache Society
dfaI^2^	Heterogeneity statistic (percentage of variability due to heterogeneity)
JBI	Joanna Briggs Institute
k	Number of studies included in the analysis/meta-analysis
MeSH	Medical Subject Headings
N	Total sample size
n	Number of participants/cases (subsample)
NA	Not Applicable
OR	Odds Ratio
PICO	Population, Intervention/Exposure, Comparison, Outcome
PR	Prevalence Ratio
PROSPERO	International Prospective Register of Systematic Reviews
Q	Cochran’s Q statistic (heterogeneity)
REML	Restricted Maximum Likelihood
RN	Registered Nurse
RR	Risk Ratio
SWD	Shift Work Disorder (also used in some sources as Shift Work Sleep Disorder)
tiab	Title/Abstract field (PubMed search field tag)
UK	United Kingdom
YLDs	Years Lived with Disability
τ^2^	Between-study variance in meta-analysis (heterogeneity)

## Appendix A

**Table A1:** Risk-of-bias assessment of included studies (JBI Critical Appraisal Checklist for Studies Reporting Prevalence Data).

Item	Bjorvatn et al. [17]	d’Ettorre et al. [18]	Xie et al. [25]	Wang et al. [24]
1. Was the sample frame appropriate to address the target population?	Yes	Yes	Yes	Yes
2. Were study participants sampled in an appropriate way?	Yes	Yes	Yes	Yes
3. Was the sample size adequate?	Yes	Yes	Yes	Yes
4. Were the study subjects and the setting described in detail?	Yes	Yes	Yes	Yes
5. Was the data analysis conducted with sufficient coverage of the identified sample?	Unclear	Unclear	Unclear	Unclear
6. Were valid methods used for the identification of the condition?	Yes	Yes	Yes	Yes
7. Was the condition measured in a standard, reliable way for all participants?	Yes	Yes	Yes	Yes
8. Was there appropriate statistical analysis?	No	No	Unclear	Unclear
9. Was the response rate adequate, and if not, was the low response rate managed appropriately?	Unclear	No	Yes	Yes
Include	Yes	Yes	Yes	Yes
